# M. Velpeau’s Method of Treating Varicose Veins

**Published:** 1842-04-30

**Authors:** 


M. VELPEAU'S *method of treating Varicose Veins.* [From a Clinical Lecture at La Charité.]—In 1830 I first tried my operation on animals, and in 1835 I first employed it on the living subject. Since then I have repeated the same operation so frequently in public and private practice, that I am unable to tell you how often it has been performed. A strong, sharp pin, with a large head, and a waxed thread, are the only instruments that I use. The position of the patient is a matter of some importance. He must be placed in such a posture as will render the veins tumid and prominent. The trunk of the vein is now raised up with the fingers' end, the pin is passed below the ends of the nails and underneath the vein; it will be necessary to protect the finger with a thimble or a roll of linen, for the tissues under the varicose vein are often hard and very resistant. This simple process must be repeated with regard to every dilated vein; eight, ten, twelve, or fifteen pins may be required from the foot up to the knee, but, generally speaking, three or four are sufficient. When the veins are free and moveable under the skin, it is easy to pass the pins under them; but, when they are applied closely to the bones, this is sometimes impossible; you must then pass a strong pin perpendicularly downwards, and then direct it obliquely under the trunk of the vein. The pins, being all placed, are fixed with threads; at first I applied the ligatures as we do for hare lip, but this did not exercise sufficient pressure. I now twist the thread circularly round the pin, and draw it tight. The pins and ligatures are not removed before the sixth or twelfth day, when the tissues embraced between them have been destroyed. But even if the eschar be not detached at this period I remove them, because I feel confident that the vein is obliterated.

The introduction of the pin gives very little pain, but the constriction exercised by the ligature is excessively painful. Hence, you should commence with the highest pin, in order to cut off the nervous communication as much as possible. The effects of the operation are simple. The tissues embraced by the ligatures mortify and are separated, leaving a sore, which soon heals up. A hard chord forms above and below; this is the vein in process of obliteration. No dressing is required after this operation. You have merely to cut short the ends of the pins, to prevent them from pricking the patient. Unless the inflammation be severe the patient may go about; if it be excessive, then you confine him to bed, and have recourse to the antiphlogistic treatment. As soon as the eschars come away, the small sores which remain are treated as common abscesses or burns.

The operation which I have just described is extremely simple and easy of execution; the whole process consists in passing a pin and winding a bit of thread round it. It fulfils the object of obliterating the vein as well as any other method, but is free from the dangers which attend them. You may ask, however, if it always succeeds in establishing a radical cure. Our answer must be, No. The disease will not always yield. When one dilated vein is obliterated, three or four others soon appear, the superficial branches anastomose with the deep ones, and nothing is more difficult than to obstruct completely the circulation in a varicose limb. Hence the efficacy of every process or method that may be proposed for the radical cure of varix is extremely doubtful. In the observations which I have addressed to you I have considered one point only—viz., the necessity of obliterating the veins as a means of curing varix; this being admitted, we have to seek for the best means, and I think the one which I have proposed to you affords the greatest advantages. Of more than one hundred patients on whom I have operated by this method, not one has presented any dangerous symptoms; some slight external phlebitis and the formation of some small abscesses were the only consequences of any moment that ensued. Still, I always dreaded that such constant success, in this matter, would be interrupted by an unfavourable result, and my apprehensions have been unfortunately realized. The only fatal case occurred recently.

A man, 34 years of age, of good constitution and enjoying excellent health, was admitted into la Charité with an extensive varicose condition of the legs. The operation was performed in the usual way on the 4th of April. Several pins were passed under the veins, and the ligatures drawn tightly round them. The pain thus occasioned was very severe, and continued for several days; some slight inflammation set in around the pins. On the 10th two pins were removed, and on the 11th some more. Up to the 15th no untoward symptom appeared, but on the evening of that day the patient was seized with frequent shivering, nausea, and vomiting. On the morning of the 16th he was in the same state; the tongue was white; mouth dry; vomiting of green fluid; skin hot; headache and prostration; pulse quick; leg red and swollen. He was at once bled from the arm. On the 17th the vomiting ceased; there was delirium; slight stupor, pain of abdomen, and diarrhœa. The swelling of the leg extended up to the thigh; the pulse was small and frequent, and there were several livid spots on various parts of the body. On the 18th all the symptoms were aggravated, and the man sunk on the following day.

On examining the body after death, the vessels were found full of very fluid blood, but there was no trace of pus in any of them. The vein which had been operated on was not obliterated.—*Provincial Medical and Surgical Journal.* March 19th, 1842.

